# Interfacial Compatibility Evaluation on the Fiber Treatment in the Typha Fiber Reinforced Epoxy Composites and Their Effect on the Chemical and Mechanical Properties

**DOI:** 10.3390/polym10121316

**Published:** 2018-11-28

**Authors:** Samsul Rizal, Ikramullah, Deepu A. Gopakumar, Sulaiman Thalib, Syifaul Huzni, H. P. S. Abdul Khalil

**Affiliations:** 1 Department of Mechanical Engineering, Faculty of Engineering, Universitas Syiah Kuala, Darussalam, Banda Aceh 23111, Indonesia; samsul_r@yahoo.com (S.R.); sulaimanthalib@unsyiah.ac.id (S.T.); syifaul@unsyiah.ac.id (S.H.); 2 School of Industrial Technology, Universiti Sains Malaysia, Penang 11800, Malaysia; deepu1789@gmail.com

**Keywords:** Typha fiber, fiber treatment, wettability, epoxy composite, interfacial compatibility

## Abstract

Natural fiber composites have been widely used for various applications such as automotive components, aircraft components and sports equipment. Among the natural fibers *Typha spp* have gained considerable attention to replace synthetic fibers due to their unique nature. The untreated and alkali-treated fibers treated in different durations were dried under the sun for 4 h prior to the fabrication of *Typha* fiber reinforced epoxy composites. The chemical structure and crystallinity index of composites were examined via FT-IR and XRD respectively. The tensile, flexural and impact tests were conducted to investigate the effect of the alkali treated Typha fibers on the epoxy composite. From the microscopy analysis, it was observed that the fracture mechanism of the composite was due to the fiber and matrix debonding, fiber pull out from the matrix, and fiber damage. The tensile, flexural and impact strength of the *Typha* fiber reinforced epoxy composite were increased after 5% alkaline immersion compared to untreated *Typha* fiber composite. From these results, it can be concluded that the alkali treatment on *Typha* fiber could improve the interfacial compatibility between epoxy resin and *Typha* fiber, which resulted in the better mechanical properties and made the composite more hydrophobic. So far there is no comprehensive report about *Typha* fiber reinforcing epoxy composite, investigating the effect of the alkali treatment duration on the interfacial compatibility, and their effect on chemical and mechanical of *Typha* fiber reinforced composite, which plays a vital role to provide the overall mechanical performance to the composite.

## 1. Introduction

Since 1970 most fibers used in composites are synthetic fibers such as glass fibers, aramid, and carbon fibers because they have good mechanical properties [1]. However, synthetic fibers are not eco-friendly due to their non-biodegradable nature there by result in substantial environmental pollution. Therefore, the development of eco-friendly fiber reinforced composites has been attractive to scientists globally. Natural fibers are thought to be the solution to the problem because natural fibers are biodegradable and renewable. The development of several natural fiber composites using hemp, kenaf, coconut, sisal, banana, and pineapple apple fibers have been reported extensively [2,3] Natural fiber composites have been widely used for various applications such as automotive components, aircraft components and sports equipment [4]. The idea of using natural fiber as a reinforcer in a composite material is not something new. Humans have long used this idea, since the beginning of human civilization, grass and straw have been used to strengthen mud bricks. The use of natural fibers on composites can be cost-effective and light weight [5], another advantage possessed by natural fibers are their mechanical properties, which are not inferior to synthetic glass fibers that are often used as reinforcing fibers in composites [6]. One of the natural fibers that have the potential to replace synthetic fibers is *Typha spp*. *Typha* fibers are abundant in most countries [7]. *Typha* is very much grown on wetlands in the province of Aceh. This plant has been often considered a parasite because its growth dominates the wetlands. Although Typha is available abundantly in nature, its potential is still underutilized compared to other natural fibers [8]. From the previous reports it is well known that the *Typha* fiber reinforced composites have many advantages, such as good bending properties [9], being lightweight [10], having good mechanical strength, are low density and can be renewed [11]. However, so far there is no report about *Typha* fiber reinforcing epoxy composite. The objective of this paper was to investigate the effect of alkali treatment duration on the interfacial compatibility of the composite and their effect on mechanical and physical properties of *Typha* fiber reinforced composite, which plays a vital role to provide the overall mechanical performance to the composite.

In recent years, many studies have been aimed at developing biodegradable composites [12,13]. In last few decades, the natural fiber-reinforced polymer composites are starting to compete with synthetic fiber-reinforced composites such as glass fibers and carbon fibers because they have many advantages over glass fibers and carbon fibers [14]. Natural fibers have properties superior to glass fibers in terms of flexibility and stiffness [15]. Other advantages to be obtained from natural fibers compared to handmade and carbon fiber glass are their low cost, being low density and non-irritating to the skin, having reduced energy consumption, being renewable and biodegradable [16]. There are a variety of natural fibers that can be used to strengthen polymer composites, such as wood fibers, stem fibers, and leaf fibers. These fibers are widely available around the world [5].

## 2. Materials and Methods

### 2.1. Materials

*Typha* fibers were collected from swamps area in Darussalam, Aceh, Indonesia. Epoxy A331 and A062 hardener were obtained from Zarm Scientific Sdn. Bhd., Penang, Malaysia. *Typha* fiber was soaked with 5% NaOH with differential immersion duration for 1 h, 2 h, 4 h and 8 h.

### 2.2. Composite Fabrication

*Typha* fibers with 20 mm length were used as long fibers for reinforcement. The untreated and alkali-treated fibers in different treatment durations were dried under the sun for 4 h prior to the fabrication of epoxy composites. The epoxy composites were prepared in the molds with a dimension of 70 mm × 15 mm × 3 mm were cut by circular saw. *Typha* fibers of different dimensions were spread by polyester resin and epoxy by hand layup method with the composition of 40 wt % *Typha* fiber [17]. The molds were then pressed in compression molding machine at a pressure of 200 kg/cm^2^. The composites were cured for 12 h at 100 °C.

### 2.3. Chemical Composition

To investigate the chemical composition of raw *Typha* fiber and *Typha* fiber epoxy composites, FTIR analysis has been done using Shimadzu IRPrestige-21 Fourier Transform Infrared Spectroscopy (FTIR) Shimadzu Corp., Kyoto, Japan).The peaks were recorded at a wavelength of 400 to 4000 cm^−1^. to study the functional group and phase of *Typha* fiber and *Typha* fiber composites.

### 2.4. Crystalline Structure

In order to obtain the crystal structure, the wide-angle X-ray diffraction spectrum of the raw *Typha* fiber and *Typha* fiber epoxy composites were recorded on a Rigaku Dmax 2500 diffractometer (Rigaku Corp., Tokyo, Japan) The system has a rotating anode generator with a copper target and wide-angle powder goniometer. All samples were scanned in the 2θ range varying from 5° to 50°.

### 2.5. Tensile, Flexural and Impact Test

The testing of tensile strength, ductility and modulus of *Typha* fiber reinforced composite were performed via tensile test machine using a MTS Landmark (MTS System Corp., Minnesota, USA) equipped with a load cell of 10 N, with a cross-head speed of 2 mm/min according to ASTM D638-02a. The flexural strength of the composite was tested according to ASTM D790-03. The impact test of composite was conducted according to ASTM D 256-97 by Ray-Ran test equipment.

### 2.6. Morphology of the Composite

The fractured surface of the composite was observed via Scanning Electron Microscope (Evo MA10, (Carl Zeiss, Oberkochen, Germany) after the impact test, in order to study the failure mechanism. The specimen was sputtered with gold before the operation. The composite samples were observed in the SEM at an acceleration voltage of 20 KV.

### 2.7. Wettability

The static water contact angle of the composite was measured via water drop method using KSV CAM 101 (KSV Instruments Ltd., Helsinki, Finland) optical contact angle meter to examine the surface wettability of the composite. Hypodermic syringes operated through micrometer screw control slowly dripped the water onto the composite surface. The contact angle was measured on the side of the water droplet. The image was recorded for 40 s with a speed of one frame every 10 s.

### 2.8. Interfacial Shear Strength Test

The micro-bond test was performed to evaluate the interfacial shear strength of untreated and treated *Typha* fiber reinforced epoxy composite. A micro-droplet epoxy resin was dropped on *Typha* fibers with an embedded of length about 1.6–2.2 mm and fiber diameter of 0.2–0.5 mm. After the epoxy was cured, the specimens were tested on a Hegewald & Peschke inspect micro S500 (Hegewald & Peschke, Nossen, Germany) or the tensile test. The blades were applied at the end of the matrix to hold the matrix, then the tip of the fiber was given the tensile load until the fiber was pulled out from the epoxy resin.

## 3. Results and Discussion

### 3.1. FTIR and XRD Analysis of the Composites

**Figure 1:**
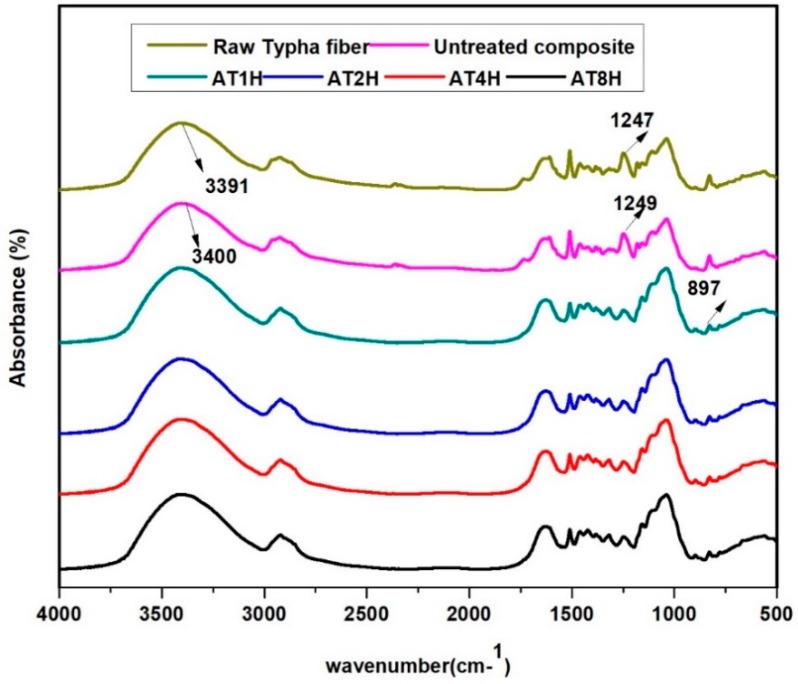
FTIR spectra of raw *Typha* fiber, untreated and treated *Typha* fiber reinforced epoxy composites.

The FTIR spectrum of raw *Typha* fiber, untreated and treated *Typha* fiber reinforced epoxy composites are shown in Figure 1. From the figure, both *Typha* fiber and untreated *Typha* fiber reinforced epoxy composite show similar spectra. The peak that emerged at 897 cm^−1^ was assigned to the C–OH denote the presence of β-glycosidic linkages between monosaccharides [18]. The peaks at 1247 and 1249 cm^−1^ were clearly seen in the spectra of raw *Typha* fiber and untreated *Typha* fiber reinforced epoxy composite respectively and which was due to the stretching of the C–O functional group indicates possible alignment of lignin. This peak was no longer present on alkali-treated *Typha* reinforced epoxy composites. The strong bands at region between 3200–3600 cm^−1^ were due to the O–H stretching [19], which were observed in all samples. The peak at 1735 cm^−1^ C=O from the stretching vibration of carboxylic acid and ester group of hemicellulose [20] appeared in both raw *Typha* fiber and untreated *Typha* fiber reinforced composite, where as in the alkali treated *Typha* fiber reinforced composites it disappeared due to the successful removal of the hemicellulose from alkali treated *Typha* fiber reinforced composites during the alkali treatment. From these results, it can be concluded that the alkali treatment had successfully removed the lignin and hemicellulose from the *Typha* fiber reinforced epoxy composite.

**Figure 2:**
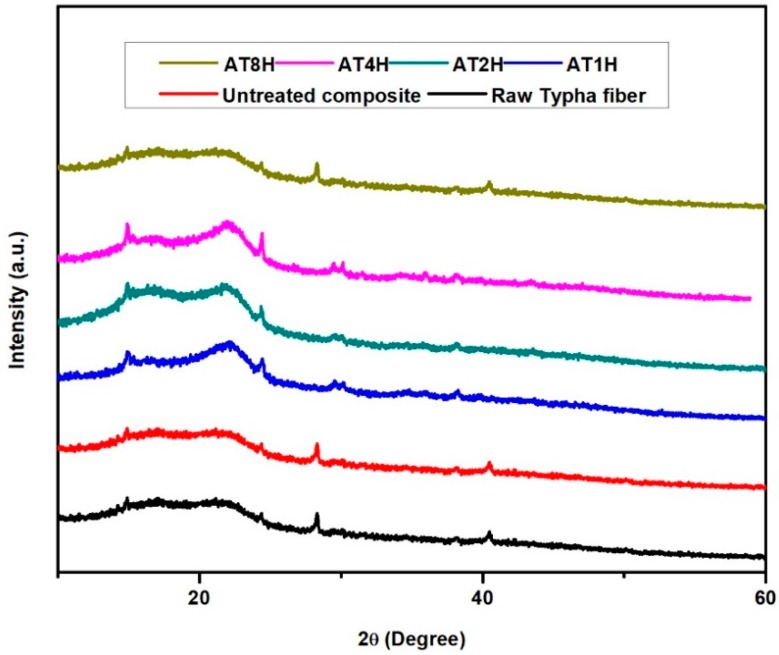
X-ray Diffraction patterns of raw *Typha* fiber, untreated and treated *Typha* fiber reinforced epoxy composites.

The XRD analysis was conducted to investigate the crystallinity index of the raw *Typha* fiber, untreated *Typha* fiber reinforced epoxy composite and treated *Typha* fiber reinforced epoxy composites. X-Ray diffraction profiles of raw Typha fiber, untreated Typha fiber reinforced epoxy composite and treated Typha fiber reinforced epoxy composites are shown in Figure 2. The crystalline index was calculated by the Equation (1):

(1) $$\mathrm{CI}=\frac{I_{002}-I_{am}}{I_{002}}\times 100$$

The intensity of the raw *Typha* fiber and untreated *Typha* fiber reinforced epoxy composites was low as compared to the alkali treated *Typha* fiber reinforced epoxy composites. This was due to the fact that during the alkali treatment, the amorphous portions like hemicellulose and lignin were removed which resulted in the enhanced peak intensity for treated *Typha* fiber reinforced epoxy composites. The crystallinity index of raw *Typha* fiber and untreated *Typha* fiber reinforced epoxy composites was 32.7% and 37.5% respectively. Meanwhile, the crystallinity index of the composites which were reinforced with 5% alkali-treated *Typha* fiber with duration of 1, 2, 4 and 8-h were 42.9%, 43.8%, 44.2%, and 36.5%, respectively. It is well known that higher crystallinity will result in good mechanical properties. This enhanced crystallinity index in the *Typha* fiber reinforced epoxy composite was due to the removal of amorphous portions like hemicellulose and lignin from the *Typha* fibers during the alkali treatment. From these results, it can be concluded that the crystallinity index of the *Typha* fiber reinforced epoxy composite was increased after the 5% alkaline treatment of the *Typha* fibers.

### 3.2. Interfacial Shear Strength Evaluation of the Composites

The interfacial shear strength of untreated and 5% alkali treated *Typha* fiber reinforced epoxy composites are shown in Table 1. From Table 1, it is clearly shown that the average IFSS value for untreated *Typha* fiber reinforced epoxy composite was 2.24 MPa, whereas in the case of alkali treated *Typha* fiber reinforced epoxy composites, the value was higher than the untreated *Typha* fiber reinforced epoxy composite. The average IFSS value of alkali treated *Typha* fiber reinforced epoxy composites with treatment durations of 1, 2, 4 and 8 h were 2.718, 3.753, 3.96 and 4.185 MPa respectively. From these results, it can be concluded that the alkali-treated *Typha* fiber had good interface adhesion with the epoxy matrix when compared to untreated *Typha* fiber. This enhanced interface adhesion in alkali treated *Typha* fiber reinforced epoxy composite was due to the successful removal of amorphous portions like hemicellulose, lignin and pectin from the *Typha* fibers during the alkali treatment [18]. Epoxy resin could penetrate into the holes, valves and cracks of the alkali treated *Typha* fiber’s surface which enables the mechanical interlocking of the epoxy matrix on the surface of the alkali treated *Typha* fiber, and thereby resulting in good bonding between the *Typha* fiber and epoxy matrix [19].

### 3.3. Tensile Strength Analysis of the Composites

**Figure 3:**
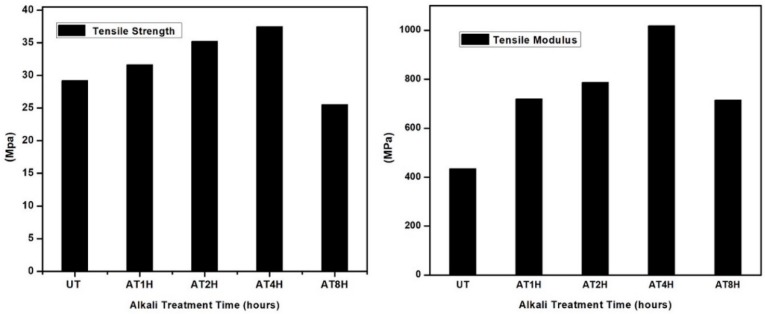
Tensile strength and Modulus of treated and untreated *Typha* fiber reinforced epoxy composite.

Figure 3 shows the *Typha* fiber reinforced epoxy composite tensile strength and modulus, with the composition of 40 wt % *Typha* fiber. It can be observed from Figure 3 that the tensile strength and modulus of the *Typha* fiber reinforced epoxy composite were increased after 5% alkaline immersion compared to untreated *Typha* fiber composite. Composite specimens with four hours of alkali treatment showed the highest tensile strength had a value of 37.4 MPa, while the untreated *Typha* composite only had 29.2 MPa. Similar studies have been reported by Cai et al. and they showed that the tensile strength of the composite were increased after the alkaline treatment of the fiber [18]. The researchers reported that the tensile behavior of composites, which were reinforced with alkali treated natural fibers, showed an enhancement in tensile strength and tensile modulus of the composites compared to natural fibers without the treatment [20,21]. Tensile strength and modulus increased proportionally with the duration of alkali treatment of fibers, but the tensile strength and modulus of composites displayed a significant decrease after an alkaline treatment of 8 h. This was due to the degradation of cellulosic molecules after the prolonged treatment with alkali. The decline in the mechanical properties, especially the tensile strength and modulus of *Typha* fibers, was reported by our previous study, in which *Typha* fibers were damaged due to the prolonged treatment with NaOH. During the alkali treatment, hemicellulose, lignin and pectin are successfully eliminated when the treatment proceeds from 1 to 8 h. There was a decrement in the tensile strength of the alkali treated *Typha* fiber reinforced epoxy composites at 8 h of treatment. However, the interfacial shear strength (IFSS) between alkali treated *Typha* fibers and epoxy matrix increased (as given in Table 1), even at 8 h of alkali treatment, which was due to the mechanical interlocking of the epoxy matrix on the alkali treated *Typha* fiber, thereby resulting in the good bonding between the epoxy matrix and the alkali treated *Typha* fiber.

### 3.4. Flexural Strength Analysis of Composites

**Figure 4:**
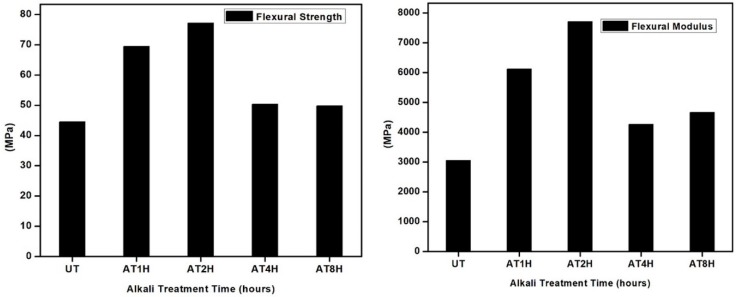
Flexural and Modulus Elasticity of untreated and treated *Typha* fiber reinforced epoxy composite.

Figure 4 shows the flexural strength and flexural modulus of alkali treated *Typha* fiber reinforced epoxy composite and untreated *Typha* fiber reinforced epoxy composite. From the figure it was clear that the flexural strength of untreated *Typha* fiber reinforced epoxy composite was around 44.5 Mpa, where as in the incase of alkali treated *Typha* fiber reinforced epoxy composite, the flexural strength was 69.5, 77.2, 50.3 and 49.8 Mpa at varying durations of immersion in NaOH for 1, 2, 4 and 8 h respectively. From these results, it can be concluded that the untreated *Typha* fiber was not an effective reinforcement due to the poor interfacial compatibility between untreated *Typha* fiber and epoxy matrix. As expected, composites with 5% NaOH treated *Typha* fiber have higher flexural strength than untreated *Typha* fiber composites, due to the removal of the amorphous portions like hemicellulose, lignin and wax from the *Typha* fibers during the alkali treatment and thereby resulting in the good interfacial compatibility between alkaline treated *Typha* fiber and epoxy matrix. This enhanced interfacial compatibility between the alkali treated *Typha* fiber and epoxy composite resulted in the enhanced flexural strength in the alkali treated *Typha* fiber reinforced epoxy composites, compared to the untreated *Typha* fiber reinforced epoxy composites. A similar observation was reported by Goud et al. and Boopathi et al., they reported that the enhancement in mechanical properties of composites was due to the good interfacial compatibility between the alkali treated fiber and matrix interface after the alkali treatment [22,23]. However, there was a decrease in the flexural strength for the 4 h alkaline treated composites. The decrease in flexural strength at the duration of alkali treatment for 4 and 8 h could be due to the degradation of the cellulose molecular structure during the prolonged treatment with the alkali, which resulted in the decreased flexural strength and flexural modulus of the composite [20,24].

### 3.5. Impact Strength Analysis of Composites

**Figure 5:**
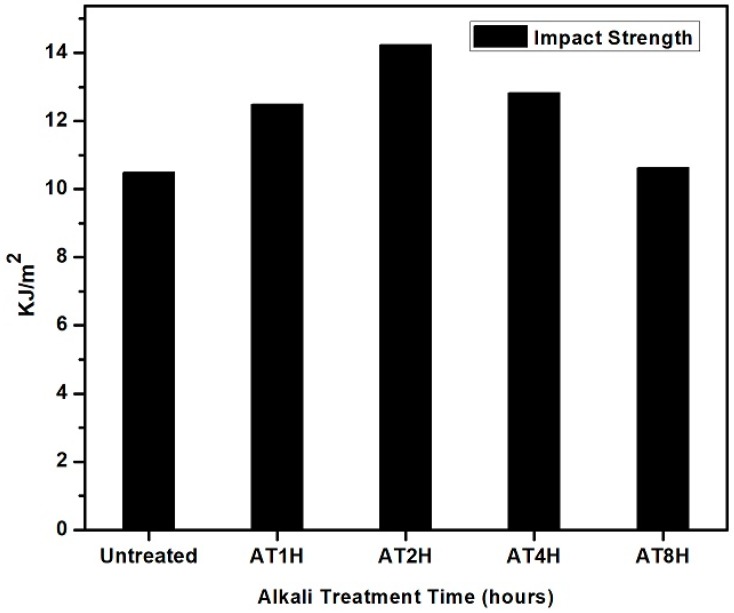
Impact strength of untreated and treated *Typha* fiber reinforced epoxy composite.

Alkaline-treated *Typha* fiber reinforced composites have better impact strength performance than untreated *Typha* fiber reinforced composites as shown in Figure 5. It was clearly shown that, the *Typha* fiber reinforced epoxy composite at 1 and 2 h alkali treated showed the highest impact strength of 12.4 and 14.2 KJ/m^2^ respectively. However, in the case of the *Typha* fiber reinforced epoxy composite at 4 and 8 h, they showed a decrease in the impact strength of 12.8 and 12.8 KJ/m^2^ respectively. This decrease in the impact strength was due to the degradation of the cellular molecular structure during the prolonged alkaline treatment. The impact strength performance of natural fiber reinforced composites is mainly influenced by several main factors such as fiber properties, matrix properties, and fiber and matrix interface properties. The composites with weak interfacial compatibility can result in poor mechanical properties of the composite, which is due to crack propagation at the matrix interface. Weak interfacial compatibility in composites could accelerate matrix crack propagation and thereby result in the debonding of fibers and matrices. The fiber/matrix interface conditions might be also affected the energy absorption in the composite. If the composite has a good interfacial compatibility, the impact load received by the epoxy matrix could be transferred to the fiber. Therefore, the fiber plays a major role as a matrix reinforcement. The impact failure on the composite could be due to the fiber damage, matrix damage, fiber pull-out from the matrix and debonding between the fiber and matrix. When the load transferred to the fiber exceeds the fiber and matrix interface strength, debonding would occur [25]. From these results it can be concluded that, the presence of alkali treated *Typha* fiber could improve the interfacial compatibility between the epoxy resin and *Typha* fiber, and thereby enhance the impact strength of the epoxy resin. In this work, it was shown that epoxy resin becomes more resistant to impact loads after reinforcing with alkaline treated *Typha* fiber. The alkali treatment of the natural fiber could improve the impact strength of the epoxy matrix when compared to the carbon/fiber epoxy composite and glass/fiber epoxy composite as shown in Table 2.

### 3.6. Scanning Electron Microscope Analysis

**Figure 6:**
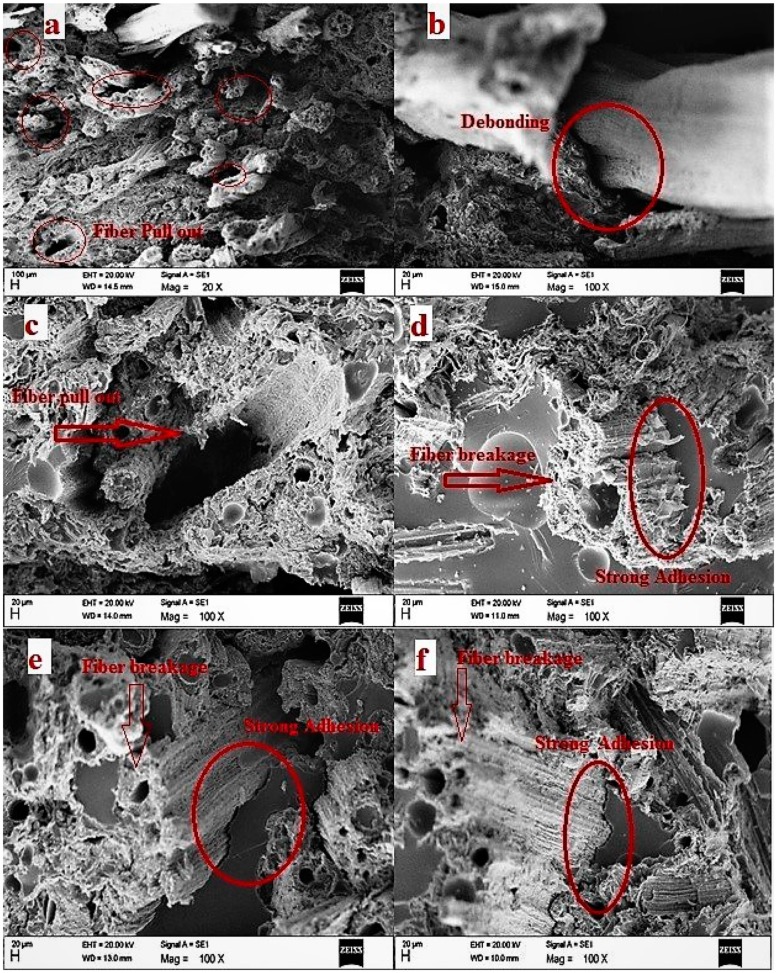
(**a**–**c**) shows the SEM images of untreated *Typha* fibers, (**d**–**f**) shows fractured surfaces of the alkali treated *Typha* fiber reinforced epoxy composites with a duration of 2, 4 and 8 h respectively.

Fractured surfaces of the specimens after impact testing were observed via scanning electron microscope to investigate the mechanism of the composite failure. Figure 6a–c shows the SEM images of untreated *Typha* fibers. From the Figure 6a–c, it can be clearly seen that, a lot of fibers were pulled out from the untreated *Typha* fiber reinforced epoxy composite which resulted in the formation of the cavities in the epoxy matrix. This was due to the poor adhesion between untreated *Typha* fiber and the epoxy matrix. Failure of composites with untreated *Typha* fiber was caused due to the poor interface interaction between untreated *Typha* fiber and epoxy matrix. In contrast, as shown in the Figure 6d, the alkali-treated *Typha* fiber reinforced epoxy composites showed a good bonding of *Typha* fibers towards epoxy resin, which can be attributed to the good interfacial adhesion between the epoxy resin and alkali treated *Typha* fibers. Moreover, from Figure 6d, we could not find much fiber pull outs in alkali treated *Typha* fiber epoxy composites, which is also in good agreement with the better interfacial adhesion proposed between the alkali treated *Typha* fibers and epoxy resin. Fractured surfaces of the alkali treated *Typha* fiber reinforced epoxy composites with a duration of 2, 4 and 8 h of immersion in 5% NaOH, are shown in the Figure 6d–f, respectively. From these images, it is clearly shown that the strong interfacial adhesion between alkali treated *Typha* fiber and epoxy resin resulted in the less fiber pull outs in the Figure 6d–f. In addition to that, the alkali treated *Typha* fibers were well embedded in the epoxy matrix, so that the load can be easily transferred from the epoxy matrix to the *Typha* fiber which resulted in the increased impact strength of the epoxy matrix [28]. Stress received by the matrix could be well distributed to the fiber when the interaction between the fiber and matrix becomes good, and thereby resulted in the enhanced mechanical properties of the composite. Alkali treatment has an important role in enhancing composite interfacial compatibility because during alkali treatment of *Typha* fibers, hemicellulose, lignin and wax were removed from the fibers which makes the surface of *Typha* fibers rougher. This increase in the roughness could be resulted in the good interaction between alkali treated *Typha* fibers and epoxy resin. Similar phenomenon was reported by Mylsamy et al. who observed the mechanical properties of the alkali treated agave reinforced epoxy composite [25]. They reported that alkali-treated agave fiber had better adhesion than the untreated one. From the microscopy analysis of the fractured surface alkali treated and untreated *Typha* fiber reinforced epoxy composites, it can be concluded that the failure of the *Typha* fiber reinforced epoxy composites was mainly due to the fiber and matrix debonding, fiber pull out from the matrix, and fiber damage.

### 3.7. Wettability Studies of the Composites

**Figure 7:**
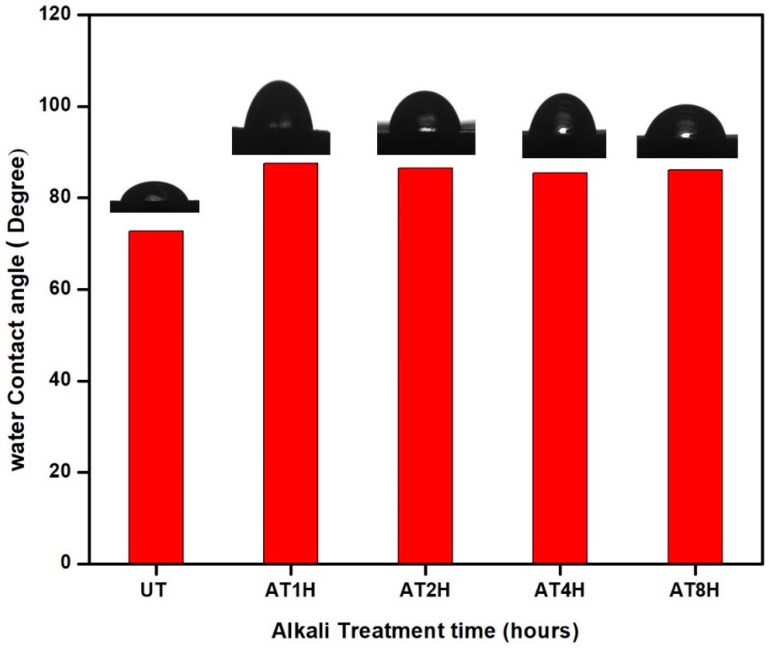
Contact angle of untreated and treated *Typha* fiber reinforced epoxy composite.

Wettability of a liquid on a solid surface can be determined using contact angle measurements. The contact angle, θ, is the angle formed by the liquid drop at the intersection point of the three-phase boundary between the plane tangent to the liquid and solid surface. A high value of θ indicates that the cohesive strength associated with bulk water is greater than the strength associated with the interaction of water with solid object surfaces, this indicates a weak interaction and poor wetting [29]. In contrast, low values of θ, with spreading tendencies in solids show strong liquid-solid interactions [30]. This shows that the forces associated with the interaction of water with a surface are greater than the cohesive strength associated with bulk liquid water [29]. Figure 7 shows water contact angles of untreated and alkali-treated *Typha* fiber reinforced composites. Composites reinforced with 5% alkaline-treated *Typha* fiber for one hour showed the highest contact angle of water at an angle of 87.50°, while the untreated composite showed the lowest contact angle. The enhancement in the contact angle for alkali treated composite was due to the enhanced surface roughness of the composite after alkali treatment [31]. The two main factors that influence the hydrophilicity of composites are surface roughness of fibers and accessibility of hydroxyl groups on the surface [32]. During alkali treatment, the hemicellulose, lignin and wax were removed which decreased the accessibility of hydroxyl groups. In addition to this, after alkali treatment the surface of the *Typha* fibers becomes rougher. This decreased accessibility of hydroxyl groups and enhanced surface roughness of the *Typha* fiber after alkali treatment resulted in the enhanced contact angle of alkali treated composite when compared to untreated one. From the contact angle studies of the treated and untreated *Typha* fiber composites, it can be concluded that the after-alkali treatment the *Typha* fiber composite became hydrophobic in nature.

### 3.8. Thermal Analysis of the Composites

**Figure 8:**
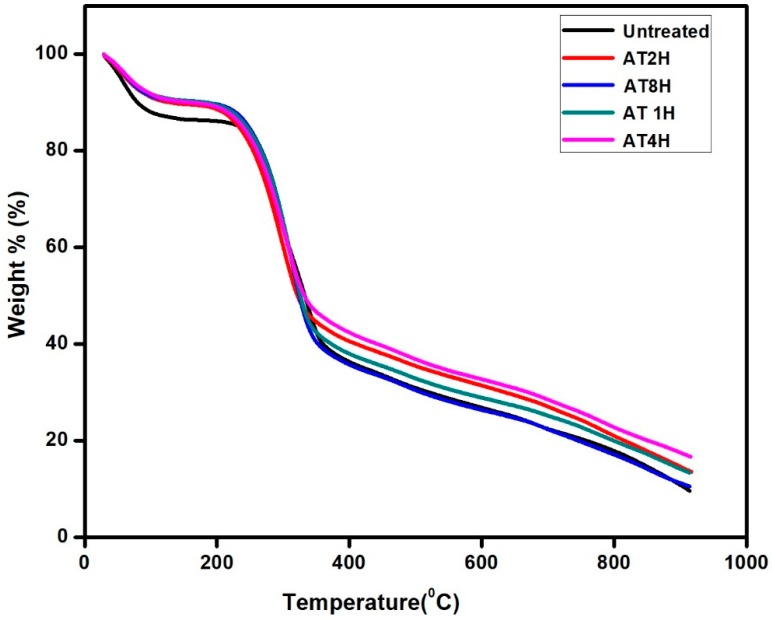
TGA curves of untreated and treated *Typha* fiber reinforced epoxy composites.

The TGA and DTG curves of untreated and treated *Typha* fiber reinforced epoxy composites are shown in the Figure 8, which clearly describes the decomposition temperature and thermal stability of the composites throughout the various duration of 5% alkali treatment. From the Figure 8, it was clearly seen that, the initial decomposition temperature (*T*_onset_) of untreated, 1, 2, 4 and 8 h alkali treated *Typha* fiber reinforced epoxy composites were found to be 244, 246, 258, 261, and 242 °C, respectively.

There was an enhancement in the initial degradation temperature of the composites reinforced with the alkali treated *Typha* fibers compared to the untreated *Typha* fiber reinforced epoxy composites, due to the removal of the amorphous portions from the *Typha* fiber during the alkali treatment. Furthermore, the value of maximum degradation temperature (*T*_max_) of untreated, 1, 2, 4 and 8 h alkali treated *Typha* fiber reinforced epoxy composites were 314, 438, 443, 445 and 435 °C, respectively. The composite treated with the 4 h alkali treatment showed the highest *T*_max_ values which was due to the highly crystalline nature of the samples. The composite treated with 4 h alkali treatment had the higher crystallinity index value of 44.2% which was higher than all the other samples, resulted in the higher thermal stability compared to the other composites. Among the alkali treated samples, the composite treated with 8h alkali treatment showed low thermal stability which was due to the degradation of the cellulose molecules during the prolonged treatment with alkali. This resulted in the low thermal stability and crystallinity index (36.5%) of the composites treated with the 8 h alkali treatment. From this thermogravimetric analysis of the treated and untreated *Typha* fiber reinforced epoxy composites data, it can be concluded that the composite treated with 4 h alkali treatment had very good thermal stability due to its high crystalline nature compared to other composites.

## 4. Conclusions

A series of alkali treated *Typha* fiber reinforced epoxy composites were fabricated via a hand layup method. Alkali treatment had been successfully done on the *Typha* fibers by immersing *Typha* fibers in 5% NaOH for 1, 2, 4 and 8 h. From the XRD and FTIR analysis, it can be concluded that the alkali treatment had been successfully removed the lignin and hemicellulose from *Typha* fiber reinforced epoxy composite and converted the amorphous *Typha* fiber into a crystalline form. The tensile, flexural, interfacial shear strength and impact strength of the *Typha* fiber reinforced epoxy composite were increased after 5% alkali immersion, compared to untreated *Typha* fiber composite. The enhancement in the mechanical properties of the alkali treated *Typha* fiber reinforced epoxy composite was due to the better interfacial compatibility between the alkali treated Typha fiber and epoxy resin. Wettability studies revealed that the alkali treated *Typha* fiber reinforced epoxy composites become more hydrophobic than the untreated one. This enhancement in contact angle for the alkali treated composite was due to the enhanced surface roughness and decreased accessibility of hydroxyl groups after the alkaline treatment. The failure mechanism of *Typha* fiber-reinforced composites was due to fiber and matrix debonding, fiber pull out from the matrix, and fiber damage. From these results it can be concluded that the alkali treatment on *Typha* fiber could improve interfacial compatibility between epoxy resin and *Typha* fiber, which results in better mechanical composite performance by making the composite more hydrophobic. We firmly believe that the demonstrated robust hydrophobic alkali treated *Typha* fiber reinforced epoxy composite will be a promising candidate for the various applications such as automotive components, aircraft components and sports equipment.

## Figures and Tables

**Table 1:** Interfacial shear strength of untreated and treated *Typha* fiber reinforced epoxy composites.

Typha Fiber Reinforced Epoxy Composites	Interfacial Shear Strength (IFSS) Mpa
Untreated	2.24
AT1H	2.718
AT2H	3.753
AT4H	3.96
AT8H	4.185

**Table 2:** Comparison of Mechanical properties of *Typha* reinforced epoxy fiber with carbon fiber/epoxy and glass fiber epoxy composites.

Fiber/Epoxy Composites	Tensile Strength (Mpa)	Flexural Strength (MPa)	Impact Strength	References
Carbon fiber/epoxy	329	525	0.4 (J/m)	Rahmai et al. (2014) [26]
Glass fiber/epoxy	179	297	1.84 (J)	Sathishkumar et al. (2014) [27]
*Typha* fiber/epoxy	37	77	14 KJ/m^2^	This work
